# QEEG characteristics associated with malnutrition-inflammation complex syndrome

**DOI:** 10.3389/fnhum.2023.944988

**Published:** 2023-02-07

**Authors:** Tirapoot Jatupornpoonsub, Paramat Thimachai, Ouppatham Supasyndh, Yodchanan Wongsawat

**Affiliations:** ^1^Brain-Computer Interface Laboratory, Department of Biomedical Engineering, Faculty of Engineering, Mahidol University, Nakhon Pathom, Thailand; ^2^Division of Nephrology, Department of Medicine, Phramongkutklao Hospital, Bangkok, Thailand

**Keywords:** quantitative electroencephalogram, malnutrition-inflammation complex syndrome, end-stage renal disease, chronic kidney disease, malnutrition-inflammation score

## Abstract

End-stage renal disease (ESRD) has been linked to cerebral complications due to the comorbidity of malnutrition and inflammation, which is referred to as malnutrition-inflammation complex syndrome (MICS). The severity of this condition is clinically assessed with the malnutrition-inflammation score (MIS), and a cutoff of five is used to optimally distinguish patients with and without MICS. However, this tool is still invasive and inconvenient, because it combines medical records, physical examination, and laboratory results. These steps require clinicians and limit MIS usage on a regular basis. Cerebral diseases in ESRD patients can be evaluated reliably and conveniently by using quantitative electroencephalogram (QEEG), which possibly reflects the severity of MICS likewise. Given the links between kidney and brain abnormalities, we hypothesized that some QEEG patterns might be associated with the severity of MICS and could be used to distinguish ESRD patients with and without MICS. Hence, we recruited 62 ESRD participants and divided them into two subgroups: ESRD with MICS (17 women (59%), age 60.31 ± 7.79 years, MIS < 5) and ESRD without MICS (20 women (61%), age 62.03 ± 9.29 years, MIS ≥ 5). These participants willingly participated in MIS and QEEG assessments. We found that MICS-related factors may alter QEEG characteristics, including the absolute power of the delta, theta, and beta 1 bands, the relative power of the theta and beta 3 subbands, the coherence of the delta and theta bands, and the amplitude asymmetry of the beta 1 band, in certain brain regions. Although most of these QEEG patterns are significantly correlated with MIS, the delta absolute power, beta 1 amplitude asymmetry, and theta coherence are the optimal inputs for the logistic regression model, which can accurately classify ESRD patients with and without MICS (90.0 ± 5.7% area under the receiver operating characteristic curve). We suggest that these QEEG features can be used not only to evaluate the severity of cerebral disorders in ESRD patients but also to noninvasively monitor MICS in clinical practice.

## 1. Introduction

Malnutrition-inflammation complex syndrome (MICS), a condition in which protein-energy malnutrition (PEM) and inflammation coexist with chronic kidney disease (CKD), has been shown to be a strong predictor of sickness, morbidity, hospitalization, and mortality in patients with end-stage renal disease (ESRD) (Kalantar-Zadeh et al., 2001). This syndrome, which has a prevalence of more than 40% (Bramania et al., 2020), requires routine monitoring to prevent undesirable outcomes. Comprehensive scoring systems, such as the subjective global assessment (SGA), dialysis malnutrition score (DMS), and malnutrition-inflammation score (MIS), are consistently recommended in clinical settings (Ho et al., 2008; Harvinder et al., 2016; Bramania et al., 2020). Unlike DMS and MIS, SGA is not invented for ESRD patients. Because laboratory results are included in MIS, this tool is more reliable than DMS. Moreover, MIS tends to be superior for predicting mortality, hospitalization, and severity of MICS in ESRD patients (Kalantar-Zadeh et al., 2001). Whereas, the total MIS score can range between 0 and 30 (ten components with each component ranging from 0 to 3), a cutoff of five has been shown to optimally distinguish between patients with and without MICS (Bramania et al., 2020). This cutoff also indicates an 88% risk of malnutrition (Harvinder et al., 2016), as well as an 80% risk of 1-year mortality in ESRD patients (Ho et al., 2008). MICS can be caused by volume overload, lack of nutrients, anorexia, and comorbid illnesses (Kalantar-Zadeh et al., 2003). These four MICS causes can be evaluated by four MIS components (Kalantar-Zadeh et al., 2001). Additional MIS components are used to assess consequences of MICS, including low functional capacity, subcutaneous fat loss, muscle wasting, low BMI, albumin level decline, and low total iron-binding capacity (TIBC) (Kalantar-Zadeh et al., 2001). The MIS, which combines medical records, physical examinations, and laboratory results, is an invasive tool that requires clinicians, limiting its routine usage. A recent study supported that MIS has very high reliability but very low daily practicability, comparing to other assessment tools for nutritional status and inflammation in CKD patients (Yamada et al., 2022). Moreover, other MICS-related factors cannot be evaluated by the MIS, including serum metabolic derangement (nitrogen, potassium, iron, prealbumin, cholesterol, and creatinine), high catabolism, endothelial damage, atherosclerosis, and uremic toxin accumulation (Kalantar-Zadeh et al., 2003). These limitations of the MIS imply that MICS assessments can be improved.

Patients with ESRD commonly present with more than one neurological complication, which has an impact on patient morbidity and mortality (Arnold et al., 2016; Hamed, 2019). Previous studies have indicated that patients with ESRD may experience brain abnormalities such as cognitive impairment, encephalopathy, seizures, asterixis, myoclonus, restless leg syndrome, extrapyramidal movement disorders, central pontine myelinolysis, depression, and stroke (Kalender et al., 2006; Baumgaertel et al., 2014; Arnold et al., 2016; Hamed, 2019). Although these cerebral complications have multiple factors, each of them can originate and progress due to MICS causes and consequences. Uremic toxin accumulation, metabolic derangement, anemia, thiamine deficiency, and secondary hyperparathyroidism are MICS-related factors and potential contributors to cerebral abnormalities in ESRD (Kalantar-Zadeh et al., 2003; Arnold et al., 2016; Hamed, 2019). This coincidence may indicate a clinical relationship between MICS and ESRD brain complications, which implies that a cerebral investigating tool may be used to predict the severity of MICS. However, in contrast to MICS assessments, there is no consensus for cerebral evaluation in ESRD patients, which may be complicated by the presence of more than one cerebral abnormality (Hamed, 2019).

Different resting-state electroencephalogram (EEG) analysis methods have been utilized to study CKD patients with cerebral disorders. These approaches include time-domain visual inspection, frequency-domain spectral analysis, quantitative EEG (QEEG), and microstate analysis (Gadewar et al., 2015; Lizio et al., 2018; Jatupornpoonsub et al., 2022). Abnormal variations in EEG amplitudes are traditionally evaluated with visual inspections. Spectral EEG analysis was later developed as a computational method in the frequency domain. Then, QEEG utilized spectral analysis values that were transformed to Z-scores by referencing a demographically (age, gender, and handedness) matched normative database (Thatcher et al., 2003). QEEG also allows neurologists to reliably and conveniently compare resting-state electrophysiological patterns between individuals (Livint Popa et al., 2020). Another method is microstate analysis, which assumes that there are quasistable EEG topographies known as microstates that represent different cognitive phenomena in the brain (Poulsen et al., 2018). Unsupervised machine learning algorithms can be used to categorize these microstate topographies in time-domain recordings into four or more groups, and each of these groups is represented by a microstate prototype. The activity of these microstate prototypes has been linked to the function of their underlying cortical networks (Mishra et al., 2020). Interestingly, the EEG findings with the aforementioned approaches have revealed complex abnormalities in the brains of patients with ESRD.

Parsons-Smith's classification (grades A–E), a resting-state EEG visual inspection criterion, has been used to evaluate the severity of encephalopathy for more than 60 years. Normal-limit EEG (grade A) is identified when suppressed alpha rhythm is frequently replaced by diffuse beta rhythm. Mild encephalopathy (grade B) is characterized by theta-disrupted alpha rhythm in both hemispheres. Moderate encephalopathy (grade C) is characterized by diffuse theta waves and random delta waves. Severe encephalopathy (grades D and E) is characterized by diffuse asynchronous theta and delta waves with or without triphasic waves (Amodio et al., 1999). These changes have been widely observed in ESRD patients (Baumgaertel et al., 2014; Gadewar et al., 2015; Arnold et al., 2016). However, compared between spectral and visual EEG analyzes, the authors found that the relative power of the theta and delta bands (spectral analysis) was significantly correlated with psychometric variations, while the quantitative visual inspection was not correlated. Thus, EEG spectral analysis may reduce the subjectivity of the interpreter and provide more reliable parameters than visual inspection (Amodio et al., 1999). Another study with ESRD patients showed that the absolute and relative power is significantly higher in the delta band and lower in the beta 1 band than in healthy controls. The finding on delta dominance was linked to uremic encephalopathy, depression, and cognitive impairment; however, the reason for the reduction in beta 1 relative power was not clearly discussed (Lai et al., 2016). QEEG variations in cognitive impairment in CKD patients are also characterized by increased power in the widespread delta and theta bands as well as reduced power in the posterior alpha and/or beta bands (Lizio et al., 2018). Unlike Alzheimer's disease, which is induced by neurodegenerative processes and characterized by alpha disappearance, cerebrovascular problems may induce cognitive impairment in ESRD, which mainly affects cortical neural synchronization in the delta frequency band. Therefore, widespread delta power was suggested as a distinctive marker of the early stage of cerebrovascular complications in CKD patients (Lizio et al., 2018). Recently, a study of the relationship between microstate EEG and MICS revealed that the frontoparietal microstate was considerably deviated in ESRD patients with more severe MICS, suggesting that abnormalities in the neural networks underlying this microstate may cause cognitive impairment (Jatupornpoonsub et al., 2022), which may also affect QEEG. As evidenced by the above studies, QEEG can be used for ESRD cerebral assessment and may be correlated with MICS severity. Thus, we used QEEG to evaluate ESRD patients in this study.

Here, we aimed to define the QEEG characteristics associated with MICS. We allocated the ESRD patients with and without MICS into two groups, ESRD-H (high risk or with MICS) and ESRD-L (low risk or without MICS), by using an MIS cutoff of five. Hence, we hypothesized that higher absolute and relative power in the delta and theta bands and lower absolute and relative power in the beta band, which can be used to characterize encephalopathy and cognitive impairment, might be observed more in the ESRD-H group than in the ESRD-L group, indicating that the ESRD-H group might have more severe cerebral complications caused by more severe MICS. However, the alpha power might not differ because alpha variations are not associated with cognitive impairment in patients with ESRD (Lizio et al., 2018). Although EEG coherence and amplitude asymmetry are unlikely to be investigated in ESRD cerebral studies, we suspected that abnormal cortical networks might not only reflect deviations in the EEG microstate but also affect coherence or/and amplitude asymmetry in the anterior-posterior axis of the brain. Therefore, we included absolute power, relative power, coherence, and amplitude asymmetry in our analysis. We also expected the aforementioned QEEG features to be correlated with the total MIS score and thus be suitable predictors of MICS. The findings in the present study could improve both cerebral monitoring and MICS evaluation in ESRD patients.

## 2. Materials and methods

### 2.1. Participants

We reviewed all related methods used in our previous study (Jatupornpoonsub et al., 2022). The experimental protocols that involved participants in this study were approved by the Institutional Review Board of Phramongkutklao Hospital, with certificate of approval (COA) number S072h/62, and the Institutional Review Board of Mahidol University, with COA number MU-CIRB 2020/393.2511. ESRD patients who underwent peritoneal dialysis (PD) or machine hemodialysis (MHD) were recruited and provided written informed consent before enrollment. All MHD patients received dialysis treatment 3–4 times per week, and PD patients received dialysate drainage every 4 h. Because abnormal EEG variants can be found in ESRD patients without any distinct cerebral diseases (Gadewar et al., 2015), we recruited patients without any history of neurological or psychiatric disease, who may especially receive benefit from EEG observations. Participants were excluded if their EEGs contained excessive artifacts, or if they recently received or had a history of exposure to central nervous system drugs, such as antiepileptic and antidepressant drugs. Although 65 patients were enrolled, the QEEG data of one woman with MICS and two men without MICS were excluded due to noise contamination. Therefore, we analyzed only 62 participants in this study.

### 2.2. MICS assessment

We used MIS questionnaires to assess the MICS status of participants. This tool has four sections: medical history, physical examination, body mass index (BMI), and a blood test. The medical history section includes the following information: (1) dry weight alterations after dialysis for 3–6 months; (2) dietary intake; (3) gastrointestinal symptoms; (4) daily functional capacity; and (5) major comorbid conditions, including duration (years) of dialysis. The physical examination section includes (6) the loss of subcutaneous fat and (7) muscle wasting. The final two sections are (8) BMI and laboratory tests, including (9) serum albumin and (10) serum total iron binding capacity (TIBC) levels. Each component has four severity levels, which are scored from 0 to 3. The total MIS score directly indicates the severity of MICS (Kalantar-Zadeh et al., 2001; Ho et al., 2008; Harvinder et al., 2016). Although the MIS score can range from 0 to 30, the recommended cutoff between ESRD patients and MICS patients is five (Bramania et al., 2020). Therefore, our study divided participants into two subgroups: ESRD-H (MIS > 5) and ESRD-L (MIS ≤ 5). The day before participants underwent hemodialysis (for patients with MHD) or 3 h after the last dialysate drainage (for patients with PD), we collected participant blood samples, the MIS score, demographic data, and QEEG measurements.

### 2.3. EEG recording

After we assessed the MICS severity of the participants, they underwent a 6 min eyes-closed EEG recording session on the same day. Due to the COVID-19 pandemic, the participant completed the recording while wearing a surgical mask. To obtain neutral resting-state EEG data, we used a soundproof experimental room with white walls. The temperature of the room was controlled to 25° Celsius, and the room was illuminated with sufficient light (300 lux). Before the session began, we requested that the participants sit upright and relaxed in an ergonomic chair with both legs forward in the most comfortable position possible. The international 10–20 electrode placement system (19 channels) with reference to the left ear lobule (A1) and ground at the right ear lobule (A2) was used to record the signal. The referential EEG monopolar montage was measured with the following protocol to optimize the signal quality. We chose an elastic cap that was optimal for the participant's head, which ensured the smallest distance between the titanium nitride electrodes and the participant's scalp. After the cap was placed on the participant's had, the gap between the electrodes and the scalp was filled with conductive gel. Then, gold cup electrodes filled with conductive paste were attached to both ear lobules, and the impedance was maintained at less than 5 kΩ. Additionally, two cup electrodes were attached above the right eyebrow (positive electrode) and at the eyelid-cheek junction (reference) to obtain electrooculogram (EOG) measurements for assessing eye movement. In addition, two cup electrodes were placed on the left (positive electrode) and right (reference) wrists to record an electrocardiogram (ECG). These two signals were used to remove EEG artifacts. Then, the participants rested for 1 min in the chair and were asked to close their eyes for the 5-min recording.

The EEG, EOG, and ECG signals were synchronously measured with a Brain Master Discovery 24E amplifier at a sampling rate of 256 Hz and a 24-bit accuracy. The signals were processed with an 80 Hz low-pass filter, monitored, recorded in the European data format (EDF), and stored on a laptop computer with Brain Master Discovery software. Before the recording, the signal offset was measured on the acquisition screen and adjusted until the offset was less than ten millivolts to maximize the signal quality. During the recording period, eye blinks, muscle artifacts, and movement artifacts that affected the EEG signals and matched with the activity of participants were marked on an artifact recording form, which was later used to exclude contaminated EEG trials.

### 2.4. QEEG feature calculation

The raw EEGs (EDF files) were processed, validated, and transformed by using NeuroGuide software version 2.8.5. According to the instruction for use of this software (ANI, 2018), those calculation steps were thoroughly described hereafter. Artifact-free EEG epochs were manually selected by using the EOG recordings, ECG recordings, and artifact recording forms as a guide. We only included total 3-min duration of all epochs from 5-min recording. Each selected epoch are not < 20 s continuously without the contamination of noises or artifacts. If we could not find this continuous 20-s epoch, the EEG was excluded from analysis. The test-retest reliability (recommended ≥90%) and split-half reliability (recommended ≥95%) were calculated to validate the signal homogeneity and measure consistency as recommended in the instruction for use (ANI, 2018). The selected EEGs were then downsampled to 128 Hz. A 5th-order Butterworth bandpass filter was applied with a passband of 1–40 Hz. For the univariate analysis, the absolute and relative powers of the 19 electrode channels were used. The absolute power is the diagonal of the autospectral matrix, which is calculated by multiplying a fast Fourier transform (FFT) of the signal by its complex conjugate and dividing by the number of frequencies. The relative power is the ratio of the specific band power to the power in a band of 1–40 Hz. For the bivariate analysis, 171 nonrepeating channel pairs were utilized to compute the amplitude asymmetry and coherence. The coherence is the square of the cross-spectrum divided by the product of the two autospectra, and the amplitude asymmetry is the ratio of the difference of the spectral power in the coupled channels to the sum of the spectral power (ANI, 2018). The EEG frequency bands included in all calculations were specified as follows: delta (1.0–4.0 Hz), theta (4.0–7.0 Hz), alpha (8.0–12.0 Hz), beta (12.0–25.0 Hz), high beta (25.0–30.0 Hz), alpha 1 (8.0–10.0 Hz), alpha 2 (10.0–12.0 Hz), beta 1 (12.0–15.0 Hz), beta 2 (15.0–18.0 Hz), and beta 3 (18.0–25.0 Hz). Using a feature of NeuroGuide software, the spectral analysis values of ESRD patients were automatically transformed to Z-scores (in the range of –3.00 to 3.00) by referencing the QEEG normative database (Thatcher et al., 2003), which included the data of normative controls that were matched to ESRD patients in term of age, sex, handedness, and recording condition (eyes closed). For further statistical analysis, the Z-scores were then written in tab delimiter text (TDT) files. We used Z-scores instead of spectral analysis values in this study, because Z-scores can minimize the effect of confounding variables (Ko et al., 2021). We also can view the Z-score as an already scaled input that can be conveniently used with a classifier. Moreover, by comparing the Z-score to zero, we can simply identify the QEEG deviation from the mean of normative controls.

### 2.5. Statistical analysis

The SciPy statistics library in Python was used for descriptive and inferential statistics (Virtanen et al., 2020). The median and interquartile range (IQR) were used to describe ordinal data, while interval data were described by the mean and standard deviation. To compare the Z-score absolute and relative power, the global mean of each frequency band in each subject group was compared; if the global means were found to be significantly different, channel-wise comparisons were performed for that band, and the Z-score of each channel was compared to zero, which is the mean of the normative data. In this case, a significant difference between the mean of the subject group and the mean of the healthy controls indicates a significant deviation between the patient and healthy EEGs. A similar protocol was applied to analyze the Z-score coherence and amplitude asymmetry. We averaged the 18 channel pairs that coupled to the same channel. For example, the average Fp2 coherence is the average coherence of the FP1-FP2, F3-FP2, C3-FP2, P3-FP2, O1-FP2, F7-FP2, T3-FP2, T5-FP2, Fz-FP2, FP2-F4, FP2-C4, FP2-P4, FP2-O2, FP2-F8, FP2-T4, FP2-T6, FP2-Cz, and FP2-Pz channel pairs. Then, the average of each channel of each frequency band in each subject group was compared. If the average was found to be significantly different, channel pair-wise comparisons were performed for that band and channel. In addition, the Z-score of each channel pair was compared to zero. A permutation-based *t*-test was utilized in these independent comparisons because this test is more robust than its parametric counterparts, and it is still reliable when the normality assumption is violated (LaFleur and Greevy, 2009). We also utilized a one-sample permutation test to compare the Z-score of each channel to zero because the difference between the average Z-score of each channel and zero does not need to be represented by a *t*-value (Nichols and Holmes, 2002). A *p* < 0.05 was considered statistically significant. As the MIS data is ordinal, we used Brunner-Munzel's test to compare the scores between the groups, as this test better controls the type I error rate, as a weaker exchangeability assumption, and a similar power to the Mann-Whitney test (Karch, 2021). The correlations between the MIS, demographic characteristics, and Z-score of the QEEG features were also analyzed using Spearman's rank order correlation, in which a value of ρ (rho) >0.40 or < –0.40 was considered a moderate correlation (Schober et al., 2018). To develop an MICS prediction model, we selected significant features according to the channel-wise comparison and correlated these features with the MIS to use as predictor variables. Then, we trained and evaluated a logistic regression model by using nested cross-validation to effectively reduce model overfitting and optimize the hyperparameters (Cawley and Talbot, 2010).

## 3. Results

### 3.1. Description of demographic information

Demographic data of 62 participants are described in Table 1. To investigate the effect of MICS on the QEEG characteristics, other variables that affect the QEEG characteristics should ideally be controlled. In practice, we cannot strictly control all variables due to the limited number of participants; however, we found that the general information was almost the same in both groups. Most clinical information and laboratory results were not significantly different between the two groups. Moreover, the effects of handedness, sex, and age are reduced in the QEEG data. Thus, the QEEG features associated with MICS can be discovered with more certainty. As shown in Table 1, the ESRD-H group had significant lower weight, BMI, albumin, TIBC, and serum iron values than the ESRD-L group (−5.129 ≤ *t* ≤ −2.509, *p* ≤ 0.014).

**Table 1:** Demographic characteristics of ESRD patients.

**General information**	**Total**	**ESRD-H**	**ESRD-L**				

Number of subjects	62	29	33				
Women (%)	37 (60)	17 (59)	20 (61)				
Diabetes (%)	40 (65)	19 (66)	21 (64)				
Right handedness (%)	52 (84)	24 (83)	28 (85)				
Hypertension (%)	41 (66)	18 (62)	23 (70)				
Hyperlipidemia (%)	20 (32)	10 (35)	10 (30)				
MHD (%)	37 (60)	17 (59)	20 (61)				
**Clinical information**	**Total**	**ESRD-H**	**ESRD-L**	***t***	***p***		
Age (years)	61.23 ± 8.59	60.31 ± 7.79	62.03 ± 9.29	–0.784	0.437		
Dialysis duration (years)	7.01 ± 7.55	7.35 ± 7.83	6.71 ± 7.4	0.329	0.748		
Weight (kg)	63.03 ± 14.27	54.9 ± 12.13	70.18 ± 12.12	–4.951	< 0.001*		
BMI (kg/m^2^)	24.2 ± 3.95	21.89 ± 3.36	26.23 ± 3.3	–5.129	< 0.001*		
BUN (mg/dL)	40.7 ± 12.53	41.52 ± 12.62	39.98 ± 12.6	0.478	0.629		
Creatinine level (mg/dL)	8.51 ± 2.67	8.23 ± 2.84	8.76 ± 2.52	–0.776	0.439		
eGFR (ml/min/1.73 m^2^)	6.04 ± 1.97	6.24 ± 2.42	5.87 ± 1.47	0.737	0.468		
**Laboratory results**	**Total**	**ESRD-H**	**ESRD-L**	***t***	***p***	⇓	⇑
Albumin (mg/dL)	4.05 ± 0.62	3.85 ± 0.65	4.23 ± 0.54	–2.509	0.014*	34 : 52 : 18	0 : 0 : 0
Calcium (mg/dL)	9.04 ± 1.22	8.87 ± 1.37	9.18 ± 1.07	–1.012	0.317	40 : 55 : 27	16 : 17 : 15
Phosphate (mg/dL)	4.3 ± 0.98	4.26 ± 0.84	4.33 ± 1.1	–0.29	0.768	5 : 3 : 6	44 : 48 : 39
Sodium (mEq/L)	137.57 ± 4.11	136.96 ± 4.17	138.11 ± 4.05	–1.098	0.274	21 : 24 : 18	29 : 28 : 30
Potassium (mEq/L)	4.28 ± 0.72	4.24 ± 0.77	4.31 ± 0.69	–0.393	0.689	11 : 14 : 9	15 : 14 : 15
Chloride (mEq/L)	96.16 ± 4.19	95.72 ± 4.42	96.55 ± 4.0	–0.777	0.437	61 : 62 : 61	0 : 0 : 0
Bicarbonate (mEq/L)	26.74 ± 2.68	27.0 ± 2.78	26.51 ± 2.61	0.717	0.477	3 : 3 : 3	19 : 21 : 18
Iron (μ g/dL)	63.84 ± 17.13	57.11 ± 14.06	69.75 ± 17.58	–3.098	0.002*	42 : 52 : 33	0 : 0 : 0
TIBC (μ g/dL)	213.06 ± 33.74	198.66 ± 34.63	225.73 ± 27.7	–3.417	0.001*	77 : 79 : 76	0 : 0 : 0
Tf saturation (%)	30.42 ± 8.39	29.51 ± 8.75	31.21 ± 8.1	–0.793	0.419	26 : 31 : 21	24 : 28 : 21
Pre-albumin (mg/dL)	34.97 ± 8.27	33.29 ± 8.03	36.45 ± 8.3	–1.521	0.136	2 : 3 : 0	52 : 48 : 55

Permutation-based t tests were used to compare variables between the ESRD-H and ESRD-L groups. Significant *p*-values are indicated by an asterisk (*). We also interpreted the laboratory results and indicated the deviation from the normal range as under (⇓) or over (⇑). These findings were reported as the percentage of subjects in each group (Total: ESRD-H : ESRD-L); for example, hypoalbuminemia was observed in 34% of the participants, 52% of the participants in the ESRD-H group, and 18% of the participants in the ESRD-L group (⇓).

### 3.2. MIS findings

We summarize the descriptive and inferential statistical analyzes of the MIS results in Table 2. By using the Brunner-Munzel test, we found that most MIS components were significantly different between the two subject groups (−4.933 ≤ ^*^ ≤ −2.050, 28.00 ≤ *DoF* ≤ 57.29, *p* ≤ 0.047), and only the fifth component, the major comorbid condition assessment, was not significantly different. Because most MIS components were significantly different between the ESRD-H and ESRD-L groups, a ranking from the most to the least different MIS component could indicate the effect size of various factors on MICS. The tenth component, which indicates the serum TIBC level, is the most different factor between the groups, which is consistent with the laboratory data (Table 1). The seventh, second, and sixth components, which indicate muscle wasting, dietary intake, and loss of subcutaneous fat, were considerably different. The eighth, fourth, and third components, which indicate BMI, daily functional capacity, and gastrointestinal symptoms, were moderately different. Finally, the ninth and first components of MIS, which indicate the serum albumin level and dry weight alterations after dialysis, were almost not significantly different.

**Table 2:** The description and comparison of MIS answers in both subject groups.

**Question**	**Score frequency (%)**								**MIS**		p^^*^	**DoF**	***p***

	**ESRD-H**				**ESRD-L**				**ESRD-H**	**ESRD-L**			
	**0**	**1**	**2**	**3**	**0**	**1**	**2**	**3**					
1	76	14	3	7	94	6			0 (0, 0)	0 (0, 0)	–2.05	31.85	0.047*
2	55	45			100				0 (0, 1)	0 (0, 0)	–4.77	28	< 0.001*
3	79	21			100				0 (0, 0)	0 (0, 0)	–2.703	28	0.012*
4	72	28			97	3			0 (0, 1)	0 (0, 0)	–2.736	35.16	0.01*
5	17	42	41		3	55	42		1 (1, 2)	1 (1, 2)	0.674	51.57	0.503
6	28	48	24		70	30			1 (0, 1)	0 (0, 1)	–4.339	46.43	< 0.001*
7	24	48	28		70	30			1 (1, 2)	0 (0, 1)	–4.902	46.43	< 0.001*
8	72	7	14	7	97		3		0 (0, 1)	0 (0, 0)	–2.736	34.12	0.01*
9	45	17	38		70	15	15		1 (0, 2)	0 (0, 1)	–2.195	54.16	0.032*
10	7	31	55	7	27	64	6	3	2 (1, 2)	1 (0, 1)	–4.933	57.29	< 0.001*

The highest score frequency in each subject group is underlined. The studentized stochastic superiority statistic (p^^*^), degree of freedom (DoF), and *p*-values (p) were obtained by using the Brunner-Munzel test, which was utilized to compare the MIS between the groups. The significant *p*-values are marked with an asterisk (*). The ten MIS components include: (1) dry weight alterations after dialysis for 3–6 months; (2) quantity of dietary intake; (3) gastrointestinal symptoms; (4) daily functional capacity; and (5) major comorbid conditions, including duration (years) of dialysis; (6) loss of subcutaneous fat; (7) muscle wasting; (8) BMI; (9) serum albumin; and (10) serum total iron binding capacity (TIBC) levels.

### 3.3. QEEG spectral analysis findings

On average, the EEG epochs included in the spectral analysis had a test-retest reliability of 93.90 ± 1.97% and a split-half reliability of 97.39 ± 1.21%, suggesting that these QEEG analyzes were sufficiently reliable. In this section, we thoroughly describe the significantly different QEEG features in the ESRD-L and ESRD-H groups.

#### 3.3.1. Absolute power

**Figure 1:**
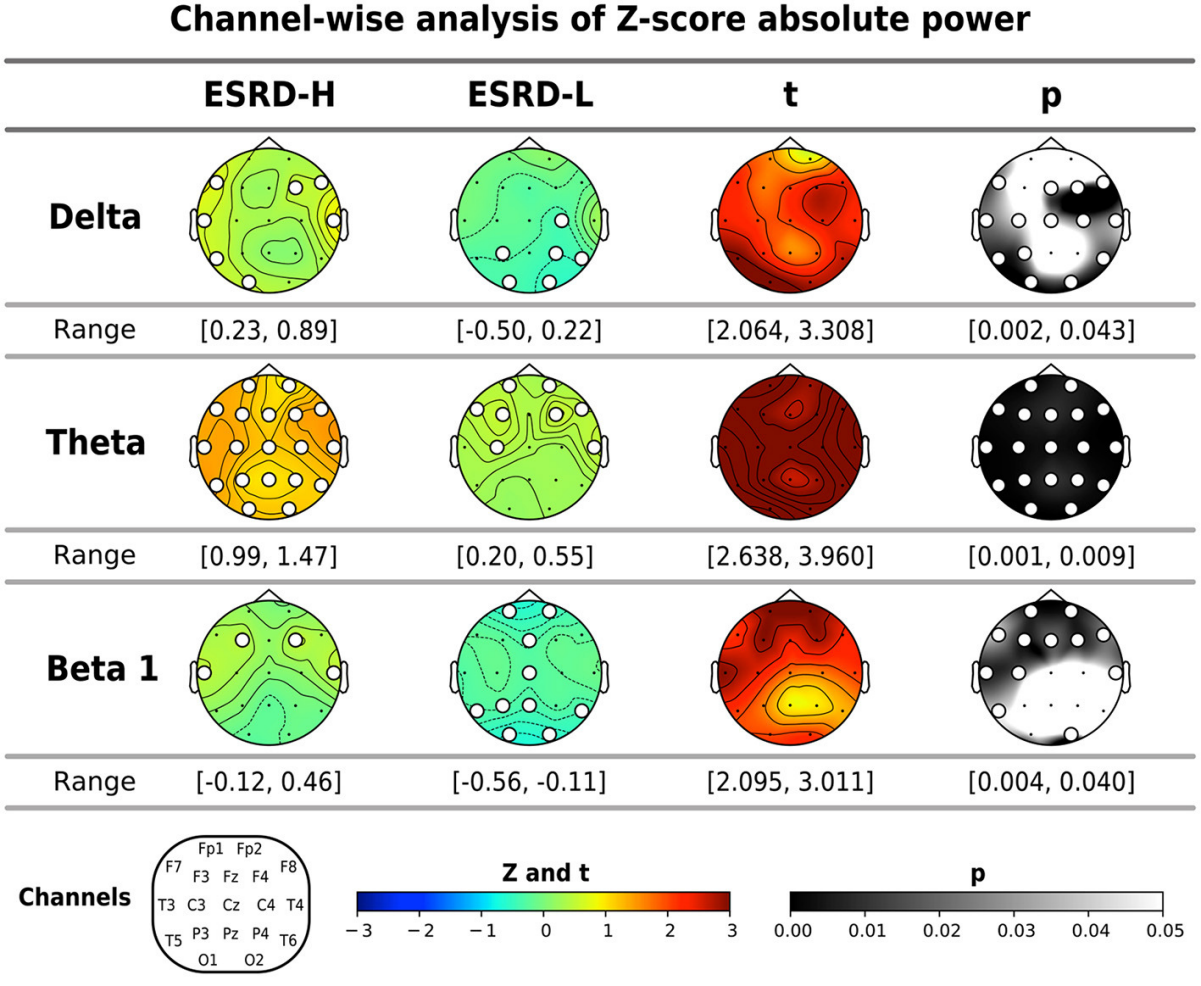
The significant band powers (delta, theta, and beta 1) according to the global mean analysis of the Z-score FFT absolute powers were determined through channel-wise comparisons between subject groups and comparisons to zero (mean of normative data) by using permutation-based *t*-tests and one-sample permutation tests. The white dots indicate significant channels in the map. All values are represented by color bars and described in the closed interval, except the *t*- and *p*-values, which indicate significant values.

The global mean of the absolute power Z-score between subject groups is shown in Table 3. We revealed that the global means of the delta, theta, and beta 1 absolute power were significantly higher in the ESRD-H group than in the ESRD-L group (2.486 ≤ *t* ≤ 3.551, 0.001 ≤ *p* ≤ 0.013). The global mean Z-scores of these frequency bands were positive in ESRD-H subjects, while ESRD-L subjects had negative global mean Z-scores in the delta and beta 1 bands and positive global mean Z-scores in the theta band. In particular, we analyzed each frequency in these bands and discovered that all frequency components differed (2.273 ≤ *t* ≤ 3.626, 0.001 ≤ *p* ≤ 0.026). Thus, the significant findings from the global mean analyzes of the delta, theta, and beta 1 band power indicate the effect of these frequencies in these bands. Furthermore, we investigated the absolute power of these bands for a channel-wise comparison, as demonstrated in Figure 1. The theta absolute power in the ESRD-H group was significantly higher than that in the ESRD-L group in all channels (2.638 ≤ *t* ≤ 3.960, 0.001 ≤ *p* ≤ 0.009). The absolute power of the delta and beta 1 bands was higher in the ESRD-H group than in the ESRD-L group in some regions. The differences in the delta absolute power were also found in other channels, with the exception of some frontal (FP1, FP2, and F3) and parietal (Pz and P4) electrodes (2.064 ≤ *t* ≤ 3.308, 0.002 ≤ *p* ≤ 0.043). For the beta 1 band absolute power, significant regions were identified in some channels in the frontal and left temporal regions, along with P3, T4, and O2 (2.095 ≤ *t* ≤ 3.011, 0.004 ≤ *p* ≤ 0.040).

**Table 3:** The global mean comparison in the univariate QEEG analysis.

**Analysis**	**Band power**	**Frequency (Hz)**	**ESRD-H**	**ESRD-L**	***t***	***p***
Average Z-score absolute power	Delta	1.0–4.0	0.41 ± 1.09	–0.21 ± 0.7	2.673	0.009
		1.0	–0.36 ± 1.0	–1.03 ± 0.52	3.384	0.001
		2.0	0.82 ± 1.2	0.21 ± 0.76	2.392	0.022
		3.0	0.69 ± 1.06	0.08 ± 0.74	2.639	0.012
	Theta	4.0–7.0	1.25 ± 1.1	0.35 ± 0.88	3.551	0.001
		4.0	0.76 ± 1.05	0.13 ± 0.72	2.778	0.008
		5.0	0.99 ± 1.09	0.23 ± 0.79	3.187	0.002
		6.0	1.44 ± 1.44	0.35 ± 0.9	3.626	0.001
		7.0	1.39 ± 1.26	0.44 ± 1.06	3.226	0.002
	Beta 1	12.0–15.0	0.15 ± 0.8	–0.32 ± 0.68	2.486	0.013
		12.0	0.07 ± 0.79	–0.34 ± 0.59	2.273	0.026
		13.0	0.16 ± 0.82	–0.3 ± 0.58	2.601	0.010
		14.0	0.24 ± 0.89	–0.29 ± 0.66	2.712	0.006
		15.0	0.21 ± 0.86	–0.25 ± 0.71	2.301	0.024
Average Z-score relative power	Theta	4.0-8.0	1.02 ± 1.44	0.23 ± 1.39	2.196	0.032
		6.0	1.08 ± 1.59	0.22 ± 1.26	2.336	0.021
		7.0	1.37 ± 1.65	0.52 ± 1.32	2.231	0.027
	Beta 3	18.0–25.0	–0.99 ± 1.26	–0.38 ± 1.0	–2.124	0.040
		20.0	–1.15 ± 1.25	–0.48 ± 1.02	–2.303	0.025
		21.0	–0.97 ± 1.22	–0.34 ± 0.98	–2.239	0.030

The significant global mean (average of 19 channels) delta, theta, and beta 1 absolute powers were found to be significantly different between groups. Each frequency in these bands was also considerably different. In contrast, some theta and beta 3 relative power frequencies (6.0, 7.0, 20.0, and 21.0 Hz) were significantly different.

By using a normative database, we can compare the Z-score of each channel to zero, which is the mean of the healthy controls. In Figure 1, the Z-score theta power of ESRD-H subjects was found to be significantly positive in all regions ($0.99\leq \bar{Z}\leq 1.47$, *p* < 0.001), whereas ESRD-L subjects exhibited positive values in only some channels ($0.34\leq \bar{Z}\leq 0.55$, 0.003 ≤ *p* ≤ 0.033). The delta and beta 1 absolute power of ESRD-L subjects mostly presented negative Z-scores in the occipital and parietal regions ($-0.59\leq \bar{Z}\leq -0.27$, 0.001 ≤ *p* ≤ 0.038); however, positive Z-scores in these bands were found in various regions in the ESRD-H group ($0.42\leq \bar{Z}\leq 0.89$, 0.001 ≤ *p* ≤ 0.045).

#### 3.3.2. Relative power

**Figure 2:**
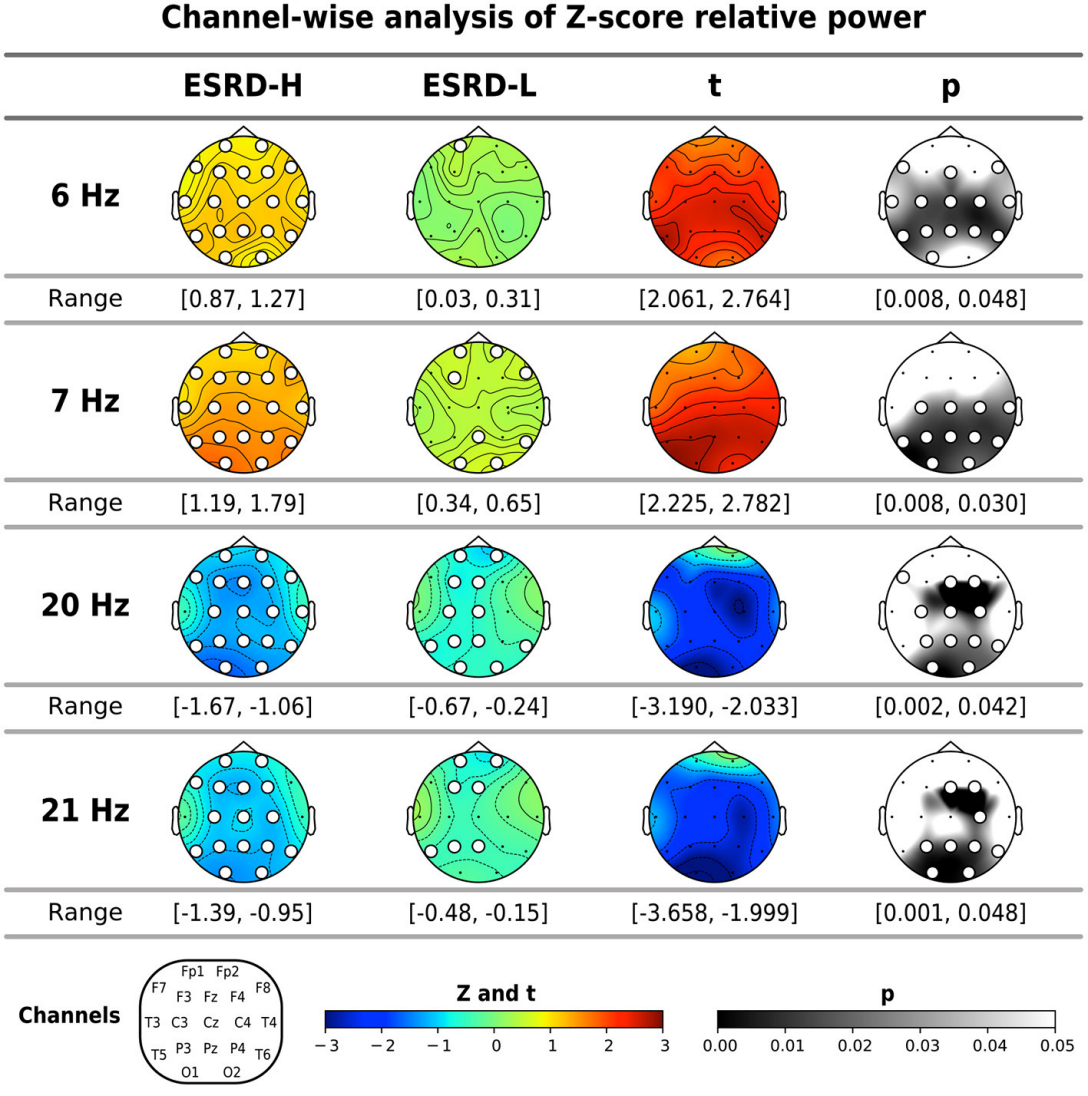
The significant frequencies (6, 7, 20, and 21 Hz) according to the global mean analysis of the Z-score FFT relative powers were determined through channel-wise comparisons between subject groups and comparisons to zero (mean of normative data) by using permutation-based *t*-tests and one-sample permutation tests. The white dots indicate significant channels in the map. All values are represented by color bars and described in the closed interval, except the *t*- and *p*-values, which indicate significant values.

The global mean Z-scores of the relative power in the theta band were positive in both subject groups, and they were significantly higher in the ESRD-H group than in the ESRD-L group (*t* = 2.196, *p* = 0.032). However, this significant difference is caused by the relative powers of 6.0 (*t* = 2.336, *p* = 0.021) and 7.0 Hz (*t* = 2.231, *p* = 0.027), as shown in Table 3. The global mean Z-scores of the relative power in the beta 3 band were negative in both groups, and they were significantly lower in the ESRD-H group than in the ESRD-L group (*t* = −2.124, *p* = 0.040). The relative power of 20.0 (*t* = −2.303, *p* = 0.025) and 21.0 Hz (*t* = −2.239, *p* = 0.030) contributed to the significant difference in beta 3 relative power. Therefore, we included the relative powers of these frequencies (6.0, 7.0, 20.0, and 21.0 Hz) in the channel-wise analysis, as shown in Figure 2. In the ESRD-H group, the Z-score relative powers of 6 and 7 Hz were more prominent in posterior regions (all except the frontal region) of the brain than other regions (2.061 ≤ *t* ≤ 2.782, 0.008 ≤ *p* ≤ 0.048). The 20 and 21 Hz relative powers of ESRD-H subjects were noticeably lower than those of ESRD-L subjects in the central, parietal, and occipital regions (−3.658 ≤ *t* ≤ −1.999, 0.001 ≤ *p* ≤ 0.048). Compared to healthy controls, the Z-score relative power of the ESRD-H group had considerably higher deviations in almost all regions. Positive deviations associated with 6 and 7 Hz appeared in all regions ($0.87\leq \bar{Z}\leq 1.79$, *p* ≤ 0.011), and negative deviations associated with 20 and 21 Hz were found in most channels ($-1.67\leq \bar{Z}\leq -0.54$, 0.001 ≤ *p* ≤ 0.048). In contrast, the Z-scores of ESRD-L subjects deviated in only some channels, as seen in Figure 2 (positive Z-score of 6 and 7 Hz: $0.41\leq \bar{Z}\leq 0.65$, 0.005 ≤ *p* ≤ 0.038, negative Z-score of 20 and 21 Hz: $-0.94\leq \bar{Z}\leq -0.42$, 0.001 ≤ *p* ≤ 0.047).

#### 3.3.3. Coherence

**Figure 3:**
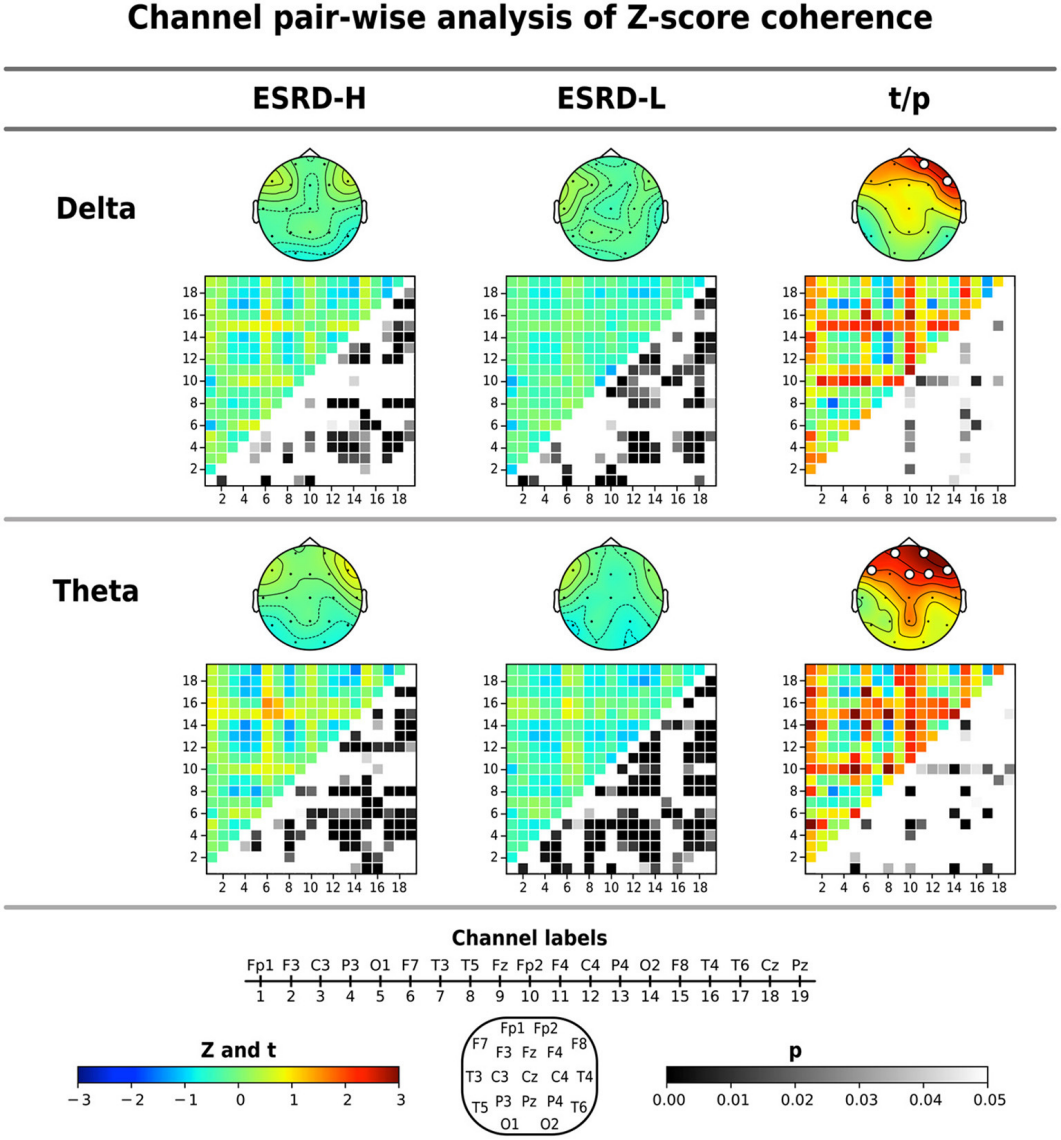
The significant findings from the global average Z-score coherence comparison were identified for each channel pair by using permutation-based *t*-tests. The average Z-score and significant channel *t*-values are shown in the topographical maps. Coherence matrices (19 x 19 channels), in which the upper triangular part indicates the *Z*- or *t* -values (171 values) and the lower triangular part signifies their corresponding *p*-values (171 values), are also shown. Note that the *p*-values of the upper triangular Z-scores were obtained through comparisons to zero using one-sample permutation tests. All values are represented by color bars, and the electrode channels are represented by numbers.

The average coherence Z-score was significantly higher in ESRD-H subjects than in ESRD-L subjects for delta and theta band frequencies, as shown in Figure 3 and Table 4. Significant differences in the average delta coherence were found in the Fp2 (*t* = 2.483, *p* = 0.015) and F8 (*t* = 2.550, *p* = 0.014) electrodes. The average Z-scores of the theta coherence differed significantly in most frontal regions, except the F3 channel (2.006 ≤ *t* ≤ 3.031, 0.004 ≤ *p* ≤ 0.049). Consequently, channel pairwise analyzes of the delta and theta coherence were performed to investigate the significant pairs (Figure 3). We found that 23 channel pairs in the delta band had significantly higher coherences in the ESRD-H group than in the ESRD-L group (2.004 ≤ *t* ≤ 2.846, 0.005 ≤ *p* ≤ 0.047). This included 9 F8 pairs coupled with central, left temporal, left occipital, F3, F7, and P4 electrodes and 12 Fp2 pairs coupled with occipital, frontal (F3, F7, Fz, F4), central (Cz, C4), parietal (P3, P4), T5, and T4 electrodes. Other 2 pairs are Fp1-O2 and F7-T4. Furthermore, we found that 29 channel pairs in the theta band had significantly higher coherences in the ESRD-H group than in the ESRD-L group (1.939 ≤ *t* ≤ 3.732, *p* ≤ 0.049), including 12 Fp2 pairs, 4 Fp1 pairs, 5 F8 pairs, 3 F7 pairs, 2 Fz pairs, F3-O1, O1-F4, and T3-T4. As shown in Figure 3, significant theta coherence was mainly found in pairs with frontal electrodes, which typical cohered with occipital electrodes and/or channels near occipital regions (T5, P3, Pz, P4, and T6).

**Table 4:** The global mean comparison in the bivariate QEEG analysis.

**Analysis**	**Band power**	**Channels**	**ESRD-H**	**ESRD-L**	***t***	***p***
Average Z-score coherence	Delta	FP2	0.32 ± 1.14	–0.29 ± 0.72	2.483	0.015
		F8	0.61 ± 1.11	0.0 ± 0.68	2.55	0.014
	Theta	FP1	0.49 ± 1.17	–0.19 ± 0.87	2.563	0.013
		F7	0.8 ± 1.25	0.26 ± 0.81	2.006	0.049
		Fz	0.10 ± 0.96	–0.39 ± 0.68	2.271	0.026
		FP2	0.55 ± 1.15	–0.29 ± 0.97	3.031	0.004
		F4	0.21 ± 1.01	–0.42 ± 0.87	2.536	0.013
		F8	0.86 ± 0.99	0.17 ± 0.82	2.916	0.004
Average Z-score amplitude asymmetry	Beta 1	C4	0.22 ± 0.47	–0.11 ± 0.52	2.533	0.013
		Cz	0.23 ± 0.69	–0.12 ± 0.46	2.337	0.021
		Pz	0.68 ± 0.8	0.13 ± 0.61	3.036	0.003

The Z-score averages of all channels (171 pairs) and each channel (18 pairs) with coherence and amplitude asymmetry were compared between subject groups by using permutation-based *t*-tests.

In the ESRD-H group, 64 channel pairs in the delta band presented both significant positive and negative coherence deviations from healthy controls. As seen in Figure 3, 21 channel pairs, including F8 pairs and Fp2 pairs, were significantly positive ($0.51\leq \bar{Z}\leq 1.14$, 0.001 ≤ *p* ≤ 0.046), while 43 channel pairs were significantly negative ($-1.19\leq \bar{Z}\leq -0.44$, *p* ≤ 0.048). However, ESRD-L subjects exhibited only negative deviations in 72 channel pairs ($-1.22\leq \bar{Z}\leq -0.34$, *p* ≤ 0.049). The Z-score theta coherence of the ESRD-H and ESRD-L patients showed significant positive and negative deviations in 75 and 100 channel pairs, respectively (Figure 3). We found 30 positive ($0.43\leq \bar{Z}\leq 1.57$, *p* ≤ 0.042) and 45 negative ($-1.50\leq \bar{Z}\leq -0.48$, *p* ≤ 0.038) channel pairs in the ESRD-H group. Moreover, we found 10 positive ($0.34\leq \bar{Z}\leq 0.90$, *p* ≤ 0.046) and 90 negative ($-1.30\leq \bar{Z}\leq -0.30$, *p* ≤ 0.044) channel pairs in the ESRD-L group.

#### 3.3.4. Amplitude asymmetry

**Figure 4:**
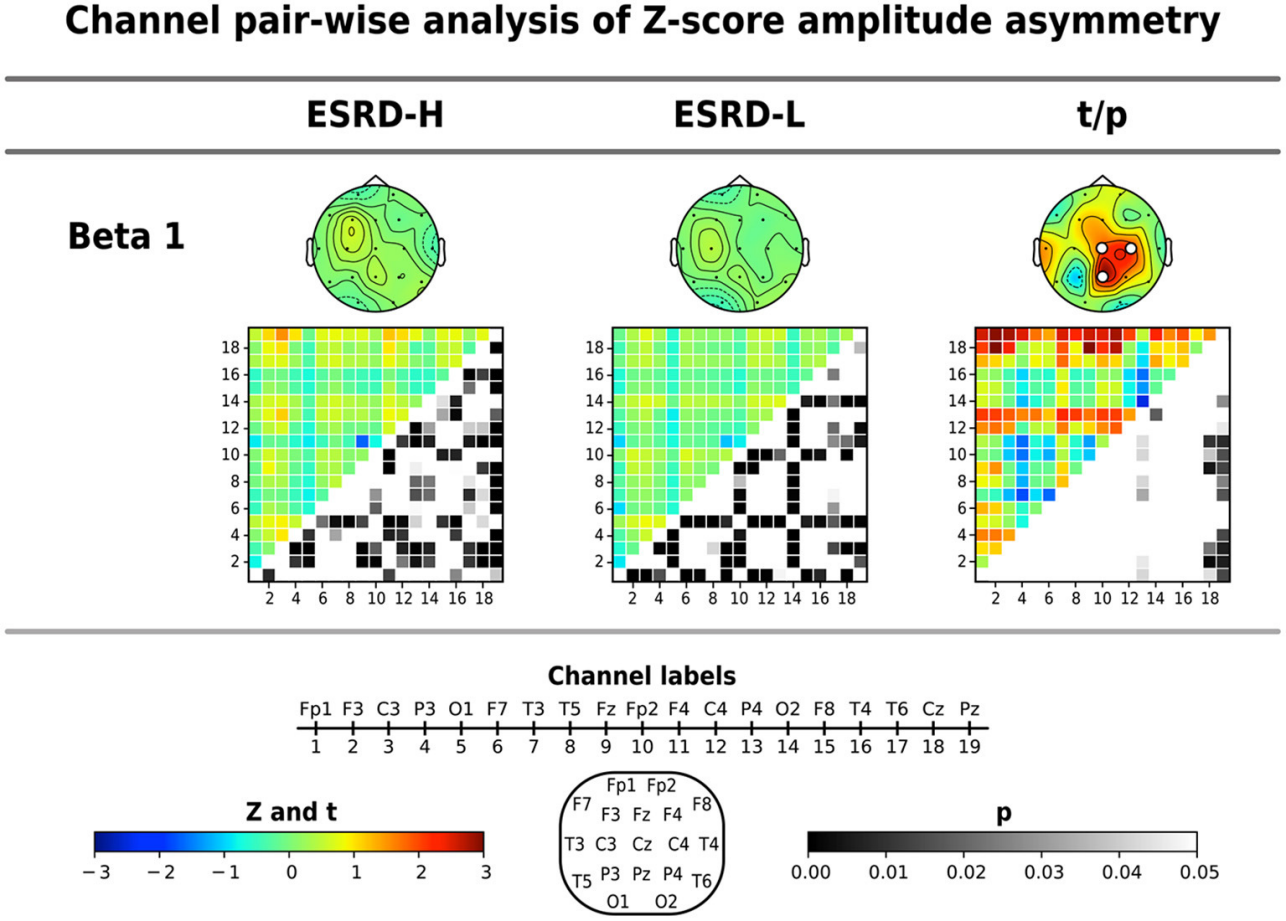
Channel pair comparisons of Z-score amplitude asymmetry were performed by using permutation-based *t*-tests. The average Z-score and significant channel *t*-values are shown in the topographical maps. The respective amplitude asymmetry matrices (19 x 19 channels), in which the upper triangular part indicates the *Z*- or *t*-values (171 values) and the lower triangular part signifies their corresponding *p*-values (171 values), are also shown. Note that the *p*-values of upper triangular Z-scores were obtained through comparisons to zero using one-sample permutation tests. All values are represented by color bars, and the electrode channels are represented by numbers.

The average Z-score of beta 1 asymmetry in C4, Cz, and Pz pairs was significantly higher in the ESRD-H group than in the ESRD-L group, as shown in Table 4 (2.337 ≤ *t* ≤ 3.036, 0.003 ≤ *p* ≤ 0.021). We also found significant differences in beta 1 asymmetry in 24 channel pairs (2.034 ≤ *t* ≤ 3.089, 0.002 ≤ *p* ≤ 0.46), including 7 P4, 6 Cz, and 11 Pz pairs (Figure 4). Beta 1 asymmetry in the P4 pairs included left temporal (T3-P4, T5-P4), frontal (Fp1-P4, Fp2-P4, F3-P4, and F4-P4), and P4-O2 asymmetry. Cz-pairs included frontal (Fp1-Cz, Fp2-Cz, Fz-Cz, F3-Cz, and F4-Cz) and C3-Cz asymmetry. Pz pairs included left temporal (T3-Pz, T5-Pz), frontal (Fp1-Pz, Fp2-Pz, Fz-Pz, F3-Pz, and F4-Pz), central (C3-Pz, C4-Pz), and O2-Pz asymmetry. The conflicting results between the C4 and P4 pairs need to be explained. In the ESRD-H group, C4 pairs typically have positive Z-scores, while P4 pairs have an equal number of positive and negative Z-scores. In the ESRD-L group, the Z-scores of both pairs are nearly zero. In contrast to the P4-pairs, the average Z-scores of the C4 pairs significantly differed; however, this difference was not due to channel-pairwise differences. Therefore, beta 1 asymmetry in P4, Cz, and Pz pairs could be used to represent different features in various subject groups.

We found that the Z-scores of 85 beta 1 asymmetry channel pairs in the ESRD-H group were significantly different from the mean of the healthy controls, including 28 negative ($-1.65\leq \bar{Z}\leq -0.26$, *p* ≤ 0.49) and 57 positive channel pairs ($0.34\leq \bar{Z}\leq 1.54$, *p* ≤ 0.44). In the ESRD-L group, we found significant deviations in 72 channel pairs, including 36 negative ($-1.10\leq \bar{Z}\leq -0.29$, *p* ≤ 0.49) and 36 positive channel pairs ($0.25\leq \bar{Z}\leq 0.85$, *p* ≤ 0.39). The Z-score beta 1 asymmetry in the P4, Cz, and Pz pairs were mainly positive in ESRD-H subjects; however, they are mostly zero in the ESRD-L group, which indicates that the P4, Cz, and Pz pair amplitudes might be lower than the corresponding pairs in the ESRD-H group, leading to higher asymmetry than observed in healthy controls.

#### 3.3.5. Distinctive QEEG characteristics of MICS

The above findings can be used to identify distinctive QEEG characteristics in the ESRD-H group. Although the QEEG spectral features of ESRD-L subjects are not completely equal to the normative mean, their overall Z-scores do not deviate considerably. In contrast to ESRD-L patients, ESRD-H subjects have Z-scores with large deviations that should be considered. In summary, MICS may lead to an excessive absolute theta power in all regions, excessive beta 1 power in frontal and left temporal regions, and excessive delta power in central, temporal, and occipital regions. The relative power of 6–7 Hz in all except frontal regions was also increased in MICS; however, the relative power of 20–21 Hz was decreased in central, parietal and occipital regions. These findings may indicate the effects of MICS on the activity of neuronal generators in posterior brain regions. High delta and theta coherence, as well as beta 1 asymmetry, could be affected by MICS. We thoroughly inspected of the channel pairwise comparison results (Figures 3, 4), and we determined that delta coherence between the right frontal (Fp2 and F8) and posterior regions of the brain was substantially increased in ESRD-H patients. Similarly, theta coherence between these frontal and posterior regions was increased. High beta 1 amplitude asymmetry between the central (P4, Pz, and Cz) and frontal regions of the brain was also observed in the ESRD-H group. Thus, we performed another regional comparison to support these subjective arguments, as shown in Table 5. Notably, beta 1 asymmetry may be caused by the slightly higher beta 1 power in the frontal region than in the central area, as shown in Figure 1. Therefore, the beta 1 absolute power and amplitude asymmetry should be interpreted simultaneously.

**Table 5:** The regional comparison in the bivariate QEEG analysis.

**Analysis**	**Band power**	**Regional pairs**		**ESRD-H**	**ESRD-L**	***t***	***p***
Average Z-score coherence	Delta	Right Frontal (Fp2 and F8)	L. temporal	0.74 ± 1.51	–0.17 ± 0.75	2.166	0.034
			R. temporal	0.35 ± 1.04	–0.0 ± 0.68	2.315	0.025
			Occipital	0.66 ± 1.58	–0.19 ± 0.78	2.193	0.031
			Central	0.41 ± 1.11	0.02 ± 0.73	2.475	0.017
			Parietal	0.6 ± 1.3	-0.06 ± 0.66	2.203	0.031
	Theta	Right Frontal (Fp2, F4, and F8)	L. temporal	0.52 ± 1.38	0.01 ± 0.75	2.293	0.019
			R. temporal	0.16 ± 1.05	–0.25 ± 0.8	2.152	0.034
			Occipital	0.42 ± 1.53	–0.5 ± 0.69	3.072	0.001
		Left Frontal (Fp1 and F7)	R. temporal	1.03 ± 1.63	0.19 ± 0.82	2.611	0.006
			Occipital	0.65 ± 1.71	–0.32 ± 0.61	3.059	0.002
Average Z-score amplitude asymmetry	Beta 1	Frontal (Fp1, Fp2, Fz, F3, and F4)	Central	0.22 ± 0.54	–0.09 ± 0.4	2.601	0.011
			Parietal	0.41 ± 0.65	0.03 ± 0.47	2.747	0.008

The Z-score regional averages of the delta and theta coherence and beta 1 amplitude asymmetry were compared between subject groups by using permutation-based *t*-tests.

Thus, we calculated the average Z-scores of the aforementioned significant channels and channel pairs in each frequency band. These values were then used in correlation analyzes with other clinical biomarkers and the MIS, which can be used to further clarify their relationship.

### 3.4. Correlation between QEEG features, clinical variables, and the MIS

**Figure 5:**
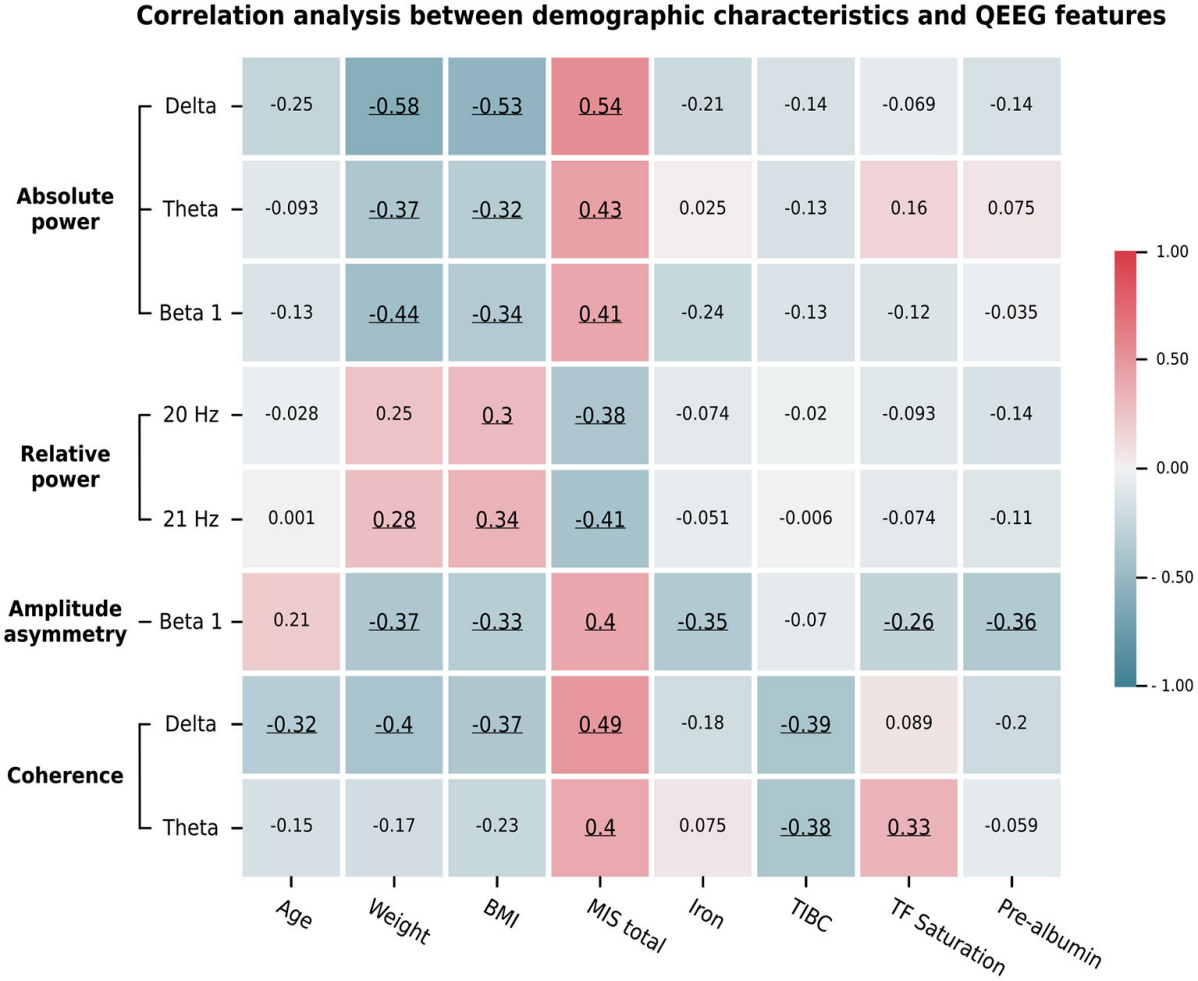
The average Z-scores of the significant QEEG features assessed by channel-wise and channel pair comparisons determined by significant correlations with the total MIS score and other demographic data. Pearson's and Spearman's rank correlation coefficients were calculated based on the data types. Their values are represented by the color bar. Only significant correlations (*p* < 0.05) are underlined.

**Figure 6:**
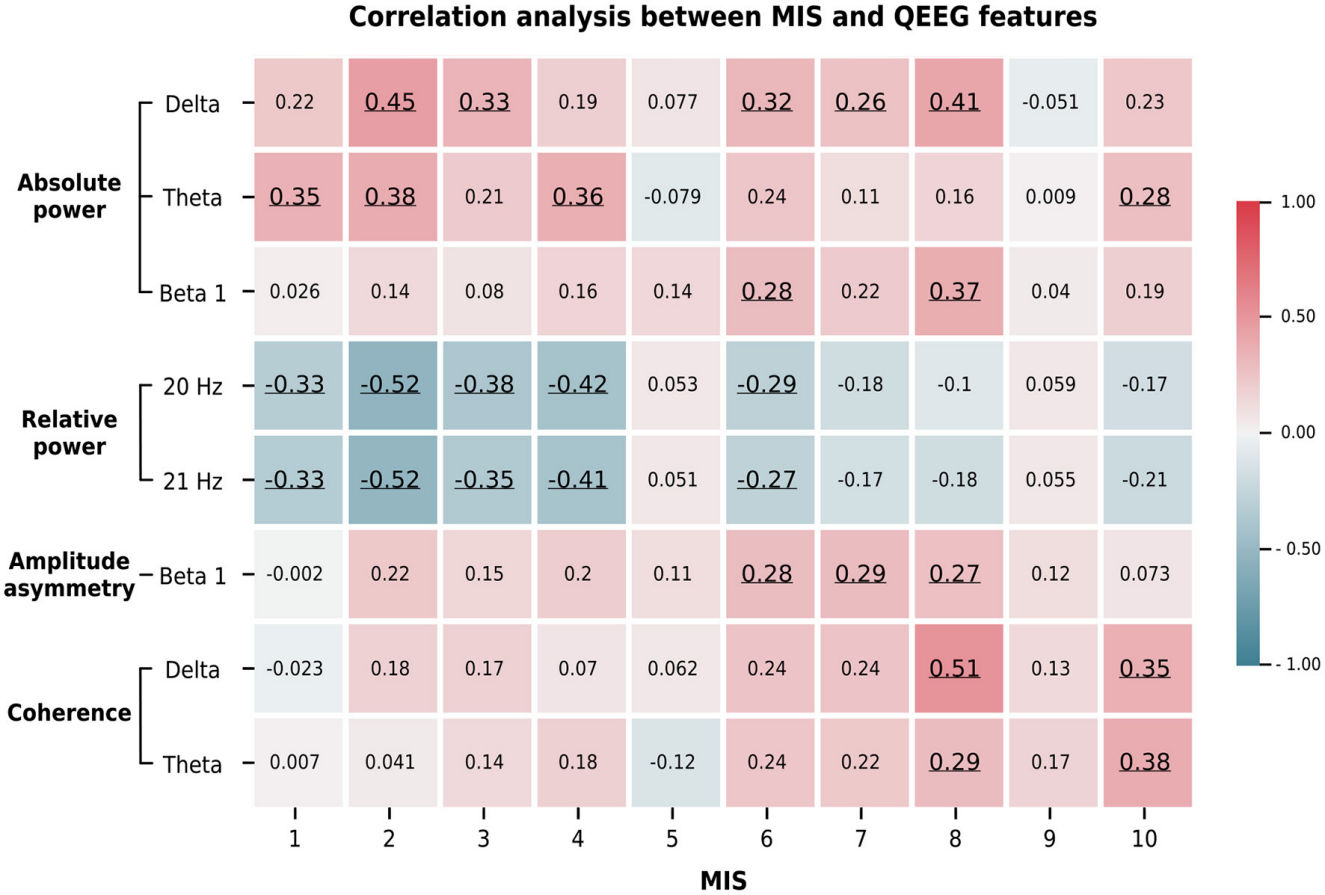
The average Z-score of the significant QEEG features correlated with the score of some MIS answers. These findings indicate the association between MICS-related factors and QEEG features. The Spearman's rank correlation coefficients are represented by the color bar. Only significant correlations (*p* < 0.05) are underlined. The ten MIS components include: (1) dry weight alterations after dialysis for 3–6 months; (2) quantity of dietary intake; (3) gastrointestinal symptoms; (4) daily functional capacity; and (5) major comorbid conditions, including duration (years) of dialysis; (6) loss of subcutaneous fat; (7) muscle wasting; (8) BMI; (9) serum albumin; and (10) serum total iron binding capacity (TIBC) levels.

We summarize the significant correlations between the averaged Z-scores of the QEEG features and the demographic data in Figure 5. The relative powers at 20 and 21 Hz were negatively and positively correlated, respectively, with the MIS and BMI score. In contrast, the absolute power in the delta, theta, and beta 1 bands showed a moderate positive correlation with the MIS and a weak to moderate negative correlation with the BMI and weight metrics. This pattern was also found in the beta 1 amplitude asymmetry and delta coherence. In addition, the beta 1 asymmetry was negatively correlated with iron and prealbumin levels, while delta coherence was negatively correlated with age and TIBC levels. In contrast, theta coherence was positively correlated with the MIS and transferrin saturation but negatively correlated with TIBC levels. Because these QEEG characteristics may be associated with MICS, we investigated which MICS consequences and causes are related to these QEEG features. Therefore, we calculated the correlation between the averaged Z-scores of the QEEG features and the score of each MIS metric, as shown in Figure 6. We found that the scores of the fifth (serum albumin level) and ninth (duration of dialysis and major comorbid diseases) MIS components were not correlated with the averaged Z-scores. However, each QEEG feature had a weak to moderate correlation with some MICS-related factor. The relative power at 20 and 21 Hz was negatively correlated with all medical examination markers, as well as subcutaneous fat loss. However, other QEEG features showed positive correlations. The delta and theta coherence were correlated with BMI and TIBC levels. The beta 1 asymmetry was correlated with subcutaneous fat loss, muscle wasting, and BMI, and the pattern was similar to that of the beta 1 absolute power. The theta absolute power was associated with dry weight changes after dialysis (3–6 months), dietary intake, daily functional capacity, and TIBC levels. The delta absolute power presented the same correlations as beta 1 asymmetry, as well as correlations with dietary intake and gastrointestinal symptoms.

### 3.5. MICS prediction with the QEEG features

The QEEG features associated with MICS were used as logistic regression predictor variables. Because all inputs were Z-scores, we did not require any scaling methods to generate our model. Table 6 shows that only the delta absolute power (*OR* = 3.717, 95*%CI* = [1.493, 9.253], *p* = 0.005), beta 1 asymmetry (*OR* = 9.116, 95*%CI* = [1.737, 47.847], *p* = 0.009), and theta coherence (*OR* = 6.501, 95*%CI* = [2.046, 20.635], *p* = 0.002) can be utilized to predict MICS in ESRD patients. When the average Z-scores of the delta absolute power, theta coherence, and beta 1 asymmetry in certain regions increased by one unit, the probability of MICS occurrence in ESRD patients increased by 4, 7, and 9 times, respectively. We found that the Newton conjugate-gradient method and L2 penalty were the optimal hyperparameters for generating this model. Nested cross-validation was applied to generate and evaluate the model. As a result, we obtained the accuracy, balanced accuracy, F1-score, AUC, precision, recall, and specificity. Although the number of ESRD-L and ESRD-H patients was imbalanced (33 vs. 29 subjects), the accuracy (80.0 ± 8.5%) and balanced accuracy (80.6 ± 9.4%) were almost equal, indicating an unbiased classification. The AUC score (90.0 ± 5.7%) also demonstrates the acceptable performance of the model. However, the standard deviation of the precision (83.0 ± 16.0%), recall (77.1 ± 17.2%), and specificity (84.0 ± 18.5%) may indicate model instability. The low recall reflects a high false-negative prediction. Although the precision was considerably high, the F1-score (76.8 ± 6.8%), which is the harmonic mean of the precision and recall, was reduced due to the low recall.

**Table 6:** Binary logistic regression and feature selection.

**Analysis**	**Features**	**Coef**.	**Std. Error**	***Z***	***p***	**95% CI**
Logistic regression with all features	Intercept	–0.724	0.851	–0.852	0.394	[–2.392, 0.943]
	Delta	2.99	1.425	2.098	0.036*	[0.197, 5.782]
	Theta	–2.198	1.089	–2.018	0.044*	[–4.332, –0.064]
	Beta 1	0.546	0.827	0.66	0.509	[–1.075, 2.167]
	20 Hz	–1.84	1.973	–0.933	0.351	[–5.706, 2.027]
	21 Hz	0.918	1.876	0.489	0.625	[–2.759, 4.594]
	Beta 1 Asymmetry	2.76	1.014	2.723	0.006*	[0.773, 4.746]
	Delta coherence	–0.101	0.737	–0.137	0.891	[–1.545, 1.344]
	Theta coherence	3.414	1.372	2.489	0.013*	[0.726, 6.103]
Logistic regression without insignificant features	Intercept	–0.57	0.718	–0.793	0.427	[–1.976, 0.837]
	Delta	2.934	1.269	2.312	0.021*	[0.446, 5.421]
	Theta	–1.356	0.906	–1.497	0.134	[–3.132, 0.42]
	Beta 1 Asymmetry	2.664	0.964	2.762	0.006*	[0.774, 4.554]
	Theta coherence	3.302	1.236	2.672	0.008*	[0.879, 5.724]
Optimal logistic regression	Intercept	–1.363	0.514	–2.652	0.008*	[–2.37, –0.356]
	Delta	1.313	0.465	2.822	0.005*	[0.401, 2.225]
	Beta 1 Asymmetry	2.21	0.846	2.612	0.009*	[0.552, 3.868]
	Theta coherence	1.872	0.59	3.174	0.002*	[0.716, 3.027]

Significant predictor variables were selected to build the optimal multiple logistic regression model, which was used to predict the probability of MICS. The coefficient (Coef.), standard error (Std. Error), and 95% confident interval of coefficient (95% CI) are used to describe the model. Wald tests were utilized to calculate the *Z*- and *p*-values, which assess the significance of the coefficients. Significant *p*-values are labeled by an asterisk (*).

## 4. Discussion

### 4.1. Remarkable findings and conflicts with the hypotheses

Significant reductions in the weight, BMI, serum albumin level, TIBC level, and serum iron level were observed in MICS patients. Most MIS components were significantly higher in ESRD patients with than in the patients without MICS. Whereas, the QEEG characteristics of ESRD-L were nearly equivalent to normative means, the QEEG characteristics of ESRD-H deviated considerably. This result was supported by the following findings. The absolute power in the delta, theta, and beta 1 bands as well as the relative power at frequencies of 6–7 and 20–21 Hz deviated mainly in posterior brain regions. The delta and theta coherence, as well as the beta 1 asymmetry, exhibit significantly high values between anterior and posterior brain regions. According to the correlation analysis, most of these QEEG characteristics, except the 6–7 Hz relative power, are correlated with the total MIS score. However, the delta absolute power, beta 1 amplitude asymmetry, and theta coherence are the optimal features for the logistic regression model, which could potentially be used to predict MICS occurrence in ESRD patients.

Previous QEEG findings in ESRD patients led us to hypothesize that compared with ESRD-L patients, higher absolute and relative power in the delta and theta bands, as well as lower absolute and relative power in the beta band, might be observed in ESRD-H patients (Amodio et al., 1999; Gadewar et al., 2015; Lai et al., 2016). However, the alpha power may not differ because alpha deviations do not characterize cognitive impairment in patients with ESRD (Lizio et al., 2018). EEG coherence or/and amplitude asymmetry in the anterior-posterior regions of the brain might deviate in the ESRD-H group due to cerebral abnormalities in these regions (Jatupornpoonsub et al., 2022). As a result, we failed to confirm some of these hypotheses. Unexpectedly, we found that the delta relative power did not differ between the ESRD-H and ESRD-L groups, while the beta absolute power was not lower but higher in the ESRD-H group than in the ESRD-L group. Slow waves (delta and theta) should not occur concurrently with fast waves (beta 1), because they represent opposing brain activities, but our results indicated the higher activity of fast and slow waves in MICS patients, which need to be explained. The high relative power at 6–7 Hz and low relative power at 20–21 Hz may be more physiologically reasonable, but this theta subbands relative power does not significantly correlate with the severity of MICS. Because the findings are complicated as seen, we provide the following thorough explanations of the findings, conflicts, limitations, and improvements.

### 4.2. QEEG characteristics associated with MICS

Based on the MIS and serum laboratory test results, ESRD patients with MICS may exhibit the following symptoms: iron deficiency, inflammation, volume retention, malnutrition, and loss of appetite. These signs can contribute to a higher risk of cerebral disorders and more severe QEEG patterns in ESRD patients with MICS (Arnold et al., 2016; Hamed, 2019). The predicted probability of MICS in ESRD patients can be suggested by the finding on a binary logistic regression model. Considering the odds ratios, when the average Z-score of the delta power, theta coherence, and beta 1 asymmetry in certain regions increased by one unit, the probability of MICS occurrence in ESRD patients increased by 4, 7, and 9 times, respectively. As discussed below, these predictor variables and other MICS-correlated QEEG features can be associated with psychiatric, motor, and cognitive disorder, which are induced by the MICS-related factors (Baumgaertel et al., 2014; Arnold et al., 2016; Hamed, 2019; Mazumder et al., 2019).

#### 4.2.1. Psychiatric aspect of MICS QEEG patterns

Between 40 and 50% of ESRD patients exhibit anorexia as a clear consequence of depression and anxiety (Carrero, 2011; Yadollahpour and Nasrollahi, 2016). A previous study found that the severity of depression was positively correlated with C-reactive protein and ferritin levels and negatively correlated with serum albumin levels, suggesting that ESRD patients with depression also have anemia and inflammation (Kalender et al., 2006). Although the pathogenic mechanisms of depression in ESRD remain unknown, self-care maintenance and inflammation are two critical consequences of this disease (Shirazian et al., 2017). Similarly, we found that the recruited ESRD-H patients had hypoalbuminemia (52%), lower dietary intake (45%), reduced appetite and occasional nausea (21%), as well as iron deficiency (low serum iron and TIBC levels). The combination of these factors may contribute to depressive QEEG characteristics. The fact that the delta, theta, and beta absolute powers are higher in depressive patients than in normal control under the eyes-closed condition has been noted in 18 publications. The authors also found that the relative powers in these three frequency bands are not significantly different (Newson and Thiagarajan, 2019). This evidence indicates that depression may affect absolute power but not relative power.

Increased delta and theta absolute power in the right hemisphere and increased theta power in the posterior cerebral areas are QEEG characteristics associated with patients with depression, and these features reflect decreased regional cortical activity in the chronic stage of depression (Grin-Yatsenko et al., 2009; Livint Popa et al., 2020). Beta power enhancement in the frontal region has been correlated with early stage of depressive or anxiety symptoms, suggesting that EEG and metabolic activities are increased in this area (Grin-Yatsenko et al., 2009). In addition, beta absolute power can be used to differentiate depressed patients and healthy controls, which patients with depression showing higher beta power than healthy subjects (Yadollahpour and Nasrollahi, 2016). In the frontal region of the brain (deep under the Fz area), researchers found that reduced rostral-dorsal anterior cingulate cortex (ACC) volume in terms of both white and gray matter causes a similar effect in depression and anxiety disorders (van Tol et al., 2010).

Because we recruited ESRD patients with no history of psychiatric disorders, the patients in this study should present early development QEEG depressive patterns. We found that the beta 1 absolute power in the frontal regions was higher in ESRD-H patients than in ESRD-L patients and healthy controls, resulting in increased beta 1 amplitude asymmetry between the frontal and central-parietal brain regions of patients with MICS. Furthermore, the regional average of the beta 1 asymmetry and absolute power is correlated with a reduction in BMI and subcutaneous fat loss, and muscle wasting is also correlated with beta 1 asymmetry. Our findings and the above evidence imply that ESRD patients that exhibit malnutrition and inflammation may be at elevated risk of developing depression and anxiety, although they did not exhibit distinct clinical signs of these diseases.

#### 4.2.2. Motor aspect of MICS QEEG patterns

Restless leg syndrome (RLS) is one of the most common movement disorders found in ESRD patients. Recently, researchers found that serum iron deficiencies may be linked to dopaminergic dysfunction in RLS (Lai et al., 2017; Lanza et al., 2017). Because cerebral iron is a cofactor for tyrosine hydroxylase, which converts tyrosine to dopamine, a lack of this enzyme can lead to dopamine deficiency in the basal ganglia, causing the sensory and motor disturbances associated with RLS (Safarpour et al., 2021). Neuroimaging studies have indicated significant deviations in the primary motor cortex and ACC of RLS patients (Lanza et al., 2017). Recently, N-acetyl-aspartate (NAA), a metabolite found in cerebral neurons that often indicates neuronal activity, was found to be higher in the dorsal ACC of RLS patients than in healthy controls (Winkelman et al., 2014). The authors assumed that high NAA levels are associated with ACC hyperactivity with this disorder. The fact that the ACC and primary motor cortex are directly connected might indicate motor hyperexcitability in patients with RLS. The overactive ACC may be related to pathogenic mechanisms of depression. Thus, many studies have found that RLS usually occurs with depression and anxiety (Saletu et al., 2002; Takaki et al., 2003; Sevim et al., 2004; Becker and Sharon, 2014). Hence, iron deficiency may contribute to the development of both early depressive symptoms and motor disturbance issues in ESRD patients with MICS. This clinical relevance is also consistent with the QEEG patterns observed in our findings.

Low-beta (12–20 Hz) activity in the parietal region was suggested to involve motor activity inhibition (Fischer et al., 2016). However, whereas normal adults generate low-beta activity to inhibit movement, patients with dopaminergic dysfunction establish low-beta activity during the resting state, which indicates that even in the resting state, the brains of these patients attempt to inhibit the motor hyperexcitability caused by dopamine deficiency (López-Azcárate et al., 2010). In addition, patients with RLS exhibited higher activity in the delta and/or theta ranges than healthy controls. The concurrence of these contrasting fast and slow wave EEGs is similar to the above-mentioned depressive patterns (Saletu et al., 2002). Because these high theta and beta activities can be alleviated in patients with RLS with dopaminergic drugs (Akpinar, 2003), we suggested that the high delta and theta waves and reduced beta 1 absolute power observed in ESRD patients with MICS may reflect abnormalities in the ACC and basal ganglia, leading to comorbidities with early depressive disorder and motor disturbances, as evidenced by the aforementioned findings.

#### 4.2.3. Cognitive aspect of MICS QEEG patterns

In this study, we measured QEEG the day before the participants underwent hemodialysis (for MHD) or 3 h after the final dialysate drainage (for PD), which increases the possibility of accumulating uremic toxins. As a result, ESRD patients with MICS clearly exhibit iron deficiency, inflammation, volume retention, and malnutrition, all of which may cause mild cognitive impairment (MCI) and early uremic encephalopathy (UE) (Hamed, 2019). MCI was found to occur in 80% of ESRD population (Hamed, 2019; Mazumder et al., 2019), and patients with adequate dialysis still present signs of MCI (Mankowska et al., 2017; Viggiano et al., 2020). Because dialysis is unlikely to alleviate MCI, many studies have suggested that cerebrovascular damage and inflammation may be the primary causes of MCI in ESRD patients (Arnold et al., 2016; Lizio et al., 2018; Hamed, 2019; Mazumder et al., 2019). UE occurs due to the accumulation of uremic toxins, and dialysis usually alleviates the symptoms of UE in ESRD patients. Therefore, MCI and UE can be clinically distinguished by the effect of dialysis. Although ESRD patients received adequate dialysis and no clinical symptoms of UE were observed, their EEG signals still deviated from those of healthy controls (Gadewar et al., 2015).

One study reported that the beta relative power was decreased in MCI patients who are more likely to develop Alzheimer's disease. However, the beta relative power of MCI patients who did not develop Alzheimer's disease was not significantly different from that of healthy controls (Baker et al., 2008). In our study, the beta 3 relative power was lower in both subject groups than in healthy controls and was even less in ESRD patients with MICS; this finding may indicate MCI in both subject groups, and comorbid MICS may increase the severity of MCI. Excessive delta and theta activity was the most common feature of both acute UE and MCI in adult ESRD patients (Amodio et al., 1999; Newson and Thiagarajan, 2019; Livint Popa et al., 2020). Higher delta activity was also a distinctive marker in the development of MCI due to cerebrovascular complications in ESRD patients (Lizio et al., 2018). Therefore, it is common to observe excessive delta and theta absolute power as well as excessive theta subband relative power in ESRD patients with MICS. However, as noted in a communication paper, EEG in encephalopathy frequently has high variability, and the average absolute power is more capable of reflecting this signal variability than the relative power (Govindan et al., 2017). The reliability of the relative power is questionable in encephalopathy classifications, and this observation may explain why the theta subband relative power result does not correlate with any clinical data and why the beta 3 relative power is not a significant predictor variable in the MICS classification model.

It has been suggested that resting-state EEG coherence in older adults decreases with increasing age (Handayani et al., 2018), indicating lower functional connectivity in the brain. However, as MCI progressed to dementia, the delta and theta coherence were both found to be significantly increased in several brain regions (Meghdadi et al., 2021). In our study, compared to healthy controls, we observed a general attenuation (most negative Z-score coherence) in the delta and theta coherence in both the ESRD-H and ESRD-L groups (Figure 3), which indicates the possibility of MCI development in these patients (Handayani et al., 2018). However, because the delta and theta coherence in the anterior-posterior axis was higher in ESRD-H patients than in ESRD-L patients, we suggested that MICS may increase the severity of MCI, leading to an increased coherence (Meghdadi et al., 2021). Because functional connectivity can reflect changes in the underlying neuronal connections (Handayani et al., 2018), an MRI study reported lower functional connectivity in ESRD patients than in healthy controls in six resting-state networks. These cortical networks include the default mode network, sensorimotor network, visual network, dorsal attention network, central executive network, and auditory network (Chen et al., 2018). According to our findings on EEG coherence and reviewed evidence, MICS comorbidity may increase the severity of MCI *via* the deviation of those resting state networks, as shown by the increased delta and theta coherence in anterior-posterior regions of the brain.

#### 4.2.4. Limitations

The following limitations should be noted. The generalizability of our findings is unknown due to the subject recruitment by convenience sampling. Because all inferences and interpretations of the QEEG characteristics are based on the evidences in previous literatures, we suggested that further research could reconfirm the discussed arguments by using neuropsychological evaluation. Although the QEEG features in the model may be physiologically explainable, the feature selection is not from a priori hypotheses, which may contribute to model overfitting. Moreover, the model may be unstable due to the small sample size (62 observations). This instability is reflected by the high standard deviations (more than 16%) of the precision, recall, and specificity. Hence, the findings of the model should be interpreted with those considerations. To improve the limitations, future research may use the discussed results to narrow the feature selection procedures and explore other sophisticated data-driven models, such as support vector machines, linear determinant analyzes, and artificial neural networks with a larger sample size. Another consideration is that we used commercial software to transform the EEG parameters to Z-scores by referencing a demographically matched normative database. On the one hand, the Z-score is considered a scaled input that can be conveniently used with a classifier. On the other hand, the use of the Z-score limits the repeatability of this study.

## 5. Conclusion

The concept of kidney-brain crosstalk has been widely studied to better understand the relationship between these two major organs in humans. Renal failure indirectly deviates the cerebral function *via* the effects of malnutrition and inflammation. By using the MIS, serum test, and QEEG, we revealed the relationship between renal disease and cerebral complications by considering MICS severity. As a result, we found that MICS-related symptoms, including iron deficiency, inflammation, volume retention, malnutrition, and loss of appetite, may contribute to cerebral complications, as reflected by deviations in the delta, theta, and beta 1 absolute powers, theta and beta 3 subband relative powers, delta and theta coherence, and beta 1 amplitude asymmetry in certain brain regions. We consequently found that only the delta absolute power, beta 1 amplitude asymmetry, and theta coherence should be used as inputs to the logistic regression model, which could potentially identify ESRD patients with and without MICS. This finding implies that QEEG characteristics may be utilized to identify MICS. Therefore, we conclude that MICS-related brain abnormalities may involve motor, psychiatric, and cognitive disturbances and that complex QEEG patterns may reflect these cerebral abnormalities. Finally, we suggest that these QEEG features can not only be used to evaluate the severity of cerebral complications in ESRD patients but they may also contribute to the noninvasive monitoring of MICS in clinical practice.

## Data availability statement

The raw data supporting the conclusions of this article will be made available by the authors, without undue reservation.

## Ethics statement

The experimental protocols that involved participants in this study were approved by the Institutional Review Board of Phramongkutklao Hospital, with certificate of approval (COA) number S072h/62, and the Institutional Review Board of Mahidol University, with COA number MU-CIRB 2020/393.2511. The patients/participants provided their written informed consent to participate in this study.

## Author contributions

TJ, PT, OS, and YW contributed to the conception and design of the study. TJ and YW performed the experiments and statistical analyzes and wrote the first draft of the manuscript. All authors contributed to revising the manuscript, read, and approved the submitted version.
